# Potential T cell epitopes of *Mycobacterium tuberculosis *that can instigate molecular mimicry against host: implications in autoimmune pathogenesis

**DOI:** 10.1186/1471-2172-13-13

**Published:** 2012-03-21

**Authors:** Sathi Babu Chodisetti, Pradeep K Rai, Uthaman Gowthaman, Susanta Pahari, Javed N Agrewala

**Affiliations:** 1 Immunology Laboratory, CSIR-Institute of Microbial Technology, Chandigarh-160036, India

## Abstract

**Background:**

Molecular mimicry between microbial antigens and host-proteins is one of the etiological enigmas for the occurrence of autoimmune diseases. T cells that recognize cross-reactive epitopes may trigger autoimmune reactions. Intriguingly, autoimmune diseases have been reported to be prevalent in tuberculosis endemic populations. Further, association of *Mycobacterium tuberculosis (M. tuberculosis) *has been implicated in different autoimmune diseases, including rheumatoid arthritis and multiple sclerosis. Although, *in silico *analyses have identified a number of *M. tuberculosis *specific vaccine candidates, the analysis on prospective cross-reactive epitopes, that may elicit autoimmune response, has not been yet attempted. Here, we have employed bioinformatics tools to determine T cell epitopes of homologous antigenic regions between *M. tuberculosis *and human proteomes.

**Results:**

Employing bioinformatics tools, we have identified potentially cross-reactive T cell epitopes restricted to predominant class I and II alleles of human leukocyte antigens (HLA). These are similar to peptides of mycobacterial proteins and considerable numbers of them are promiscuous. Some of the identified antigens corroborated with established autoimmune diseases linked with mycobacterial infection.

**Conclusions:**

The present study reveals many target proteins and their putative T cell epitopes that might have significant application in understanding the molecular basis of possible T cell autoimmune reactions during *M. tuberculosis *infections.

## Background

Although, the immune system efficiently discriminates between self and non-self, the occurrence of autoimmune diseases is a testimony to the fact that such discrimination may be imprecise [1]. Understanding the etiology of autoimmune diseases has been a great challenge to immunologists. The existence of central tolerance mechanism ensures the clonal deletion of autoreactive T cells and B cells. Nonetheless, there are ample evidences signifying that a considerable number of such cells can escape these "failsafe" mechanisms [1,2]. Immunological insults like exposure to pathogenic bacteria, viruses, aberrant expression of self proteins and exposure to cryptic antigens, etc., have been implicated to trigger and amplify the immune reactions that culminate into autoimmune diseases [3-5]. Antigenic determinants/epitopes present in pathogens, which resemble the host proteins, can potentially be a threat in activating the cells of immune system, resulting in autoimmunity [3,4]. This resemblance is popularly termed as molecular mimicry.

Many different autoimmune diseases have been hypothesized to be a result of this mistaken identity. As a result of molecular mimicry, the immune cells attack the host tissues [3,5]. The sharing of similar epitopes between the host and the pathogens may instigate autoaggression by stirring autoreactive T cells and B cells. Usually, autoreactive T cells are quiescent in the periphery, since they may recognize cryptic or low affinity epitopes. Pathogenic organisms express pathogen associated molecular patterns (PAMPs) that are perceived by the immune system as "danger signals" through Toll Like Receptors (TLRs) [6]. Hence, the "TLR licensed" antigen presenting cells (APCs) can potentially activate the self-reactive T cells, since they present antigens along with inflammatory signals. Antigenic presentation in such a context may result in high avidity interactions between autoreactive T cells and the APCs that eventually break tolerance [6]. Antigens like the pulD protein from *Klebsiella sp*., nuclear antigen-1 from Epstein-Barr virus and OSP-A from *Borrelia sp*. have been associated with diseases like ankylosing spondylitis, systemic lupus erythematosus (SLE) and Lyme arthritis, respectively [7-9]. Importantly, T cells play a pivotal role in autoimmune reactions, since they may directly attack the host tissues or help B cells to produce autoantibodies [10]. Molecular mimicry has been demonstrated in T cell specific autoimmune diseases such as multiple sclerosis (MS), myocarditis, diabetes, etc. One of the early, classic studies by Strominger's group showed that the T cells reacting to immunodominant peptide of myelin basic protein (MBP) could cross-react with viral antigens [3].

*M. tuberculosis *infects about two million people annually. In TB-endemic areas, it is estimated that almost one-third of the population is infected with *M. tuberculosis *[11]. Interestingly, an abundant presence of autoimmune diseases has been reported in these populations [12,13]. TB has been associated with many different autoimmune diseases like SLE, rheumatoid arthritis (RA), MS, etc [4,13-21]. There are ample evidences to suggest that TB reactive T cells can potentially recognize self antigens [14,17-19,21]. This has been demonstrated in animal models and in TB affected individuals. For example, T cells responding to the 65 kDa antigen of *M. tuberculosis *have been shown to be present in the synovia of arthritis patients [14,17]. Hence, during a chronic state of disease, T cells that cross-react with mycobacterial and self antigens are activated, leading to detrimental autoimmune responses. Identification of such cross-reactive epitopes may be of immense scope in understanding the pathogenesis of autoimmunity. In this era of informatics, *in silico *analyses have identified a number of *M. tuberculosis *specific T cell epitopes that could be potentially used as vaccines [22-25]. However, it warrants the information on prospective cross-reactive epitopes that may elicit autoimmune responses. Cytotoxic CD8 T cells and helper CD4 T cells recognize peptides in the context of HLA class I and class II molecules, respectively. Both the subsets have been implicated in mediating autoimmune responses. Here, we have used bioinformatics tools to identify *M. tuberculosis *and human cross-reactive T cell epitopes, restricted to predominant HLA class I and class II alleles [26,27]. Interestingly, we could identify several epitopes exhibiting similarity between human and *M. tuberculosis *proteins that may be molecular triggers of autoimmunity.

## Methods

### Alleles used in the study

Predominantly occurring MHC (major histocompatibility complex) alleles in human population for HLA class I (A*01:01, A*02:01, A*03:01, A*11:01, A*24:02, B*07:02, B*08:01) and HLA class II (DRB1*01:01, DRB1*03:01, DRB1*04:01, DRB1*07:01, DRB1*08:02, DRB1*11:01, DRB1*13:02, DRB1*15:01) were chosen for the study [26-30].

### Programs and databases

#### Netmhc2.2

NetMHC 2.2 server predicts binding of peptides to various human HLA class II alleles using artificial neural networks (ANNs) [30].

#### Netmhc3.0

NetMHC 3.0 server predicts binding of peptides to a number of different HLA class I alleles using artificial neural networks and weight matrices [30].

#### HAMAP

HAMAP (High-quality Automated and Manual Annotation of microbial Proteomes) automatically annotates a significant percentage of proteins originating from microbial genome sequencing projects [31]. HAMAP uses annotation templates for protein families to propagate annotations to all members of manually defined protein families.

#### Expasy

Expasy (Expert Protein Analysis System), is a proteomics server and allows browsing through a number of data bases as well as other cross-referenced ones [32]. It also allows access to many analytical tools for the identification of proteins, analysis of their sequence and the prediction of tertiary structure.

#### UniProt

UniProt is the world's most comprehensive catalogue of information on proteins [33]. It is a central repository of protein sequences and functions created by joining the information contained in UniProt/Swiss-Prot/TrEMBL. The UniProt knowledgebase (UniProtKB) is the central hub for the collection of functional information on proteins, with accurate, consistent and rich annotation.

### Identification and analysis of homologous *M. tuberculosis *peptides in humans

Sequences of curated proteins of *M. tuberculosis *were obtained from UNIPROT database employing HAMAP search. A total of 444 well characterized proteins of *M. tuberculosis *H37Rv were selected from the database. The four classifications of the proteins of *M. tuberculosis*, namely structural, secretory, antigenic and metabolic have been directly adopted from UNIPROT database. The proteins chosen were compared for similarity with the human proteome using BLAST program [34] from ExPASY server. Based on the BLAST results, regions of nine or more amino acids (small peptides) that were similar between the human and *M. tuberculosis *proteins were selected for further analysis. The selected human peptides were assessed for binding to different predominant HLA class I and class II alleles by using the NetMHC server. IC_50 _values were selected based on the binding scores of peptide core regions (9 amino acids length) to each allele. The peptides were classified based on predicted IC_50 _values as strong binders (IC_50 _≤ 500), weak binders (500 ≤ IC_50 _≤ 5000) and non-binders (IC_50 _≥ 5000) [35-37]. The results were then analyzed by considering the binding affinity of peptides to HLA alleles, nature of antigens, allelic associations of autoimmune diseases and tuberculosis.

## Results

### Mycobacterial proteome contains T cell epitopes that cross-react with human proteins

**Figure 1:**
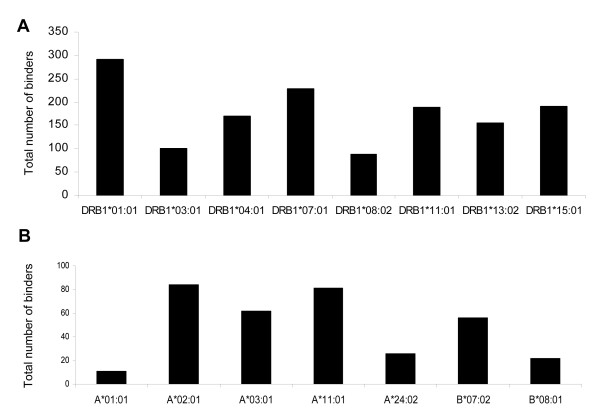
**Identification of HLA class I and II restricted T cell epitopes from host proteins that shared similarity with *M. tuberculosis* antigens**. Protein sequences of *M. tuberculosis *were subjected to BLAST search with the human proteome for identifying similarity regions. The peptides were analyzed for HLA binding for predominantly occurring HLA class I and II alleles using NetMHC server as described in methods. Peptides binding to HLA class I and class II molecules were considered to be CD8 T cell and CD4 T cell epitopes respectively. The peptides were classified based on predicted IC_50 _value as strong binders (IC_50 _≤ 500), weak binders (500 ≤ IC_50 _≤ 5000) and non-binders (IC_50 _≥ 5000). The total number of binders (strong and weak) for each allele is represented as bar diagrams for (A) HLA class II and (B) HLA class I molecules.

The analysis of the data obtained from the search between *M. tuberculosis *and human proteomes revealed considerable similarities in sequences. Many regions, that were part of metabolic proteins, were homologous (Additional file 1). A total of 460 similar regions of 9 or more amino acids were identified. We then utilized *in silico *tools to analyze the ability of these peptides to bind to different HLA class I and class II alleles. Any such common peptide that had a binding score of IC_50 _< 5000 was considered as a HLA-binder and thus was likely to be a T cell epitope. Further, peptides having an IC_50 _of ≤ 500 were considered to be strong binders [35-37]. We found considerable number of peptides (epitopes henceforth) binding to HLA class I and class II alleles (Figure 1). Our analysis revealed that the greatest number of CD4 T cell epitopes were restricted to DRB1*01:01 (291) followed by DRB1*07:01 (229) and DRB1*15:01 (191) (Figure 1). Intriguingly, DRB1*15:01 is also a susceptibility allele for tuberculosis [38,39]. The analysis of CD8 epitopes indicated maximum (84) number of peptides binding to allele A*02:01 followed by 81 for A*11:01 and 62 for A*03:01 (Figure 1).

**Figure 2:**
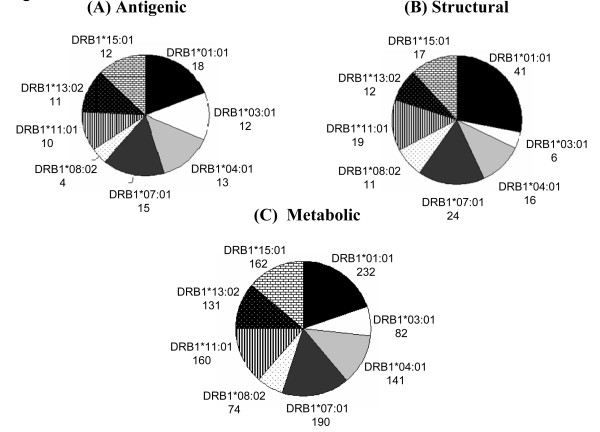
**CD4 T cell epitopes of host proteins share resemblance with different classes of *M. tuberculosis* antigens**. The absolute number of host-peptides restricted to different HLA class II alleles that showed similarity with the different classes of mycobacterial proteins is represented as pie charts for (A) antigenic; (B) structural; (C) metabolic proteins of *M. tuberculosis*.

**Figure 3:**
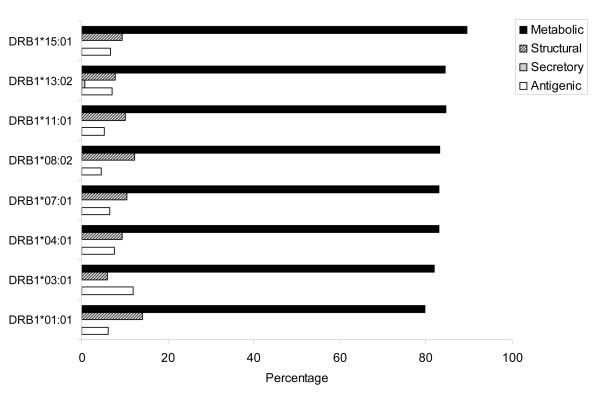
**The relative fractions of autoreactive CD4 T cell epitopes exhibiting similarity with different categories of antigens of *M. tuberculosis***. Bar diagram indicates the relative percentages of HLA class II binders from host-antigens sharing sequence corresponding to different categories of antigenic proteins of *M. tuberculosis*.

**Figure 4:**
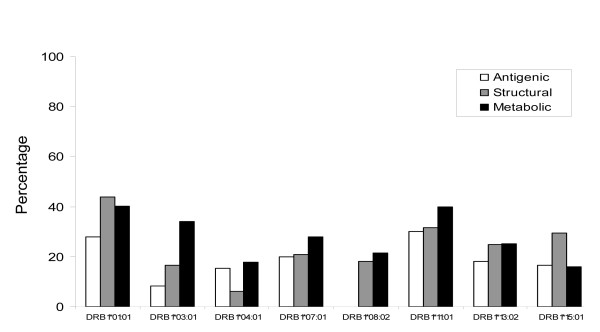
**A considerable fraction of peptides from autoantigens demonstrate strong HLA class II binding**. Bar diagram depicts the percentage of strong binders (IC_50 _≤ 500) among the total binders from host peptides that shared similarity to *M. tuberculosis *antigens.

The mycobacterial proteins for this study were classified into four different categories; antigenic, secretory, structural and metabolic proteins. The HLA binding analysis showed that the largest fraction of CD4 T cell epitopes was from metabolic proteins (Figure 2 and 3). For almost all the alleles analyzed in the current study, metabolic peptides represented > 80% of the total CD4 T cell epitopes obtained for that particular HLA allele. However, the absolute number of epitopes restricted to each allele varied greatly (Figure 2). The highest number of epitopes homologous to antigenic, structural and metabolic proteins were restricted to DRB1*01:01 (Figure 2). Interestingly, although most of the alleles had equivalent fractions of CD4 T cell epitopes from metabolic proteins, the relative representation of other categories of epitopes varied considerably (Figure 2 and 3). Highest fractions of antigenic, structural and secretory epitopes were noted for DRB1*03:01, DRB1*01:01, and DRB1*13:02, respectively (Figure 3). Next, we analyzed the fraction of strong binders among the total epitopes belonging to the different categories of proteins. We considered peptides with predicted IC_50 _value ≤ 500 as strong binders. Although, a majority of the homologous peptides were weak binders, a fraction of about 20%-40% were of higher affinities (Figure 4). We next analyzed for the presence of promiscuous epitopes among the HLA binding peptides. A peptide that could bind three or more HLA alleles was considered promiscuous. We could identify a total of 242 promiscuous peptides restricted to HLA class II molecules (Additional file 1). Interestingly, most of the promiscuous peptides (200) were from the metabolic proteins.

**Figure 5:**
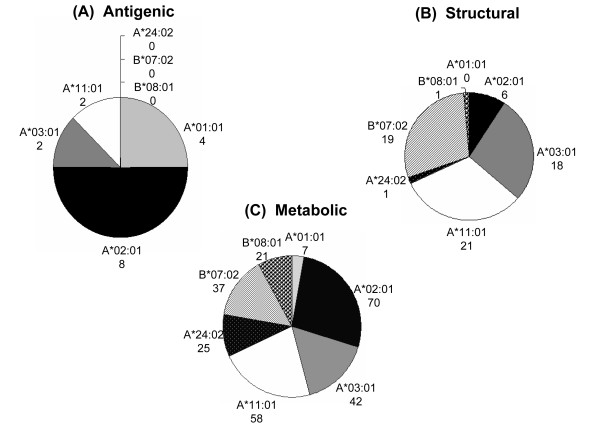
**CD8 T cell epitopes of host antigens display similarity with different classes of *M. tuberculosis* antigens**. The absolute number of host-peptides restricted to different HLA class I alleles that showed similarity with the different classes of *M. tuberculosis *proteins is represented as pie charts for (A) antigenic; (B) structural; (C) metabolic proteins.

**Figure 6:**
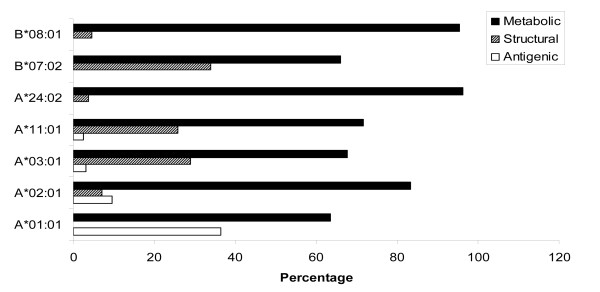
**The relative fractions of autoreactive CD8 T cell epitopes exhibiting cross-reactivity to different categories of antigens of *M. tuberculosis***. Bar diagram indicates the relative percentages of HLA class I binders of host-antigens sharing sequence similarity with the different categories of antigenic proteins of *M. tuberculosis*.

**Figure 7:**
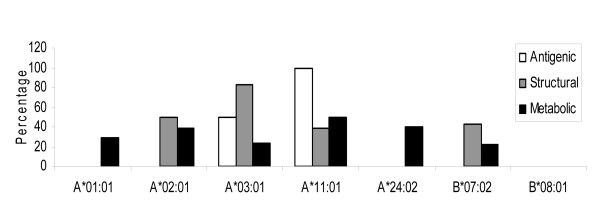
**The peptides identified from host proteins reveal strong HLA class I binding**. Bar diagram depicts the percentage of strong binders (IC_50 _≤ 500) among the total identified binders from host-peptides that shared cross-reactivity with *M. tuberculosis *antigens.

One of the most striking observations was that the total number of peptides binding to HLA class I were considerably lesser than HLA class II alleles (Figure 1). Nevertheless, the homologous peptides screened from the different classes of proteins (metabolic, antigenic, structural and secretory), were abundantly restricted to certain HLA alleles (Figure 5). For instance, allele A*02:01 bound to the highest number of antigenic (eight) and metabolic epitopes (70). Further, A*11:01 and B*07:02 indicated binding to highest numbers (21 and 19) of peptides from structural class of antigens (Figure 5). None of the HLA alleles bound to the homologous epitopes were from secretory antigens. Among the various HLA class I alleles examined, least number of CD8 epitopes exhibited binding to A*01:01 (Figure 1). Interestingly, similar to CD4 T cell epitopes, most of the peptides that were restricted to HLA class I molecules were from the metabolic proteins of *M. tuberculosis *(Figure 5 and 6). However, there was profound difference in the relative representative fractions of peptides from different classes of proteins. Peptides from all the three different classes of proteins bound to majority of the tested HLA class I or II alleles. Nevertheless, just two classes of peptides displayed binding to majority of HLA class I alleles (Figure 6). Further, we analyzed the fraction of strong binders among the total peptides for each class of proteins (Figure 7). Similar to HLA class II molecules, majority of the HLA class I alleles showed weak binding, with the exception of antigenic peptides for A*11:01 and structural peptides for A*03:01. Next we analyzed the occurrence of promiscuous CD8 T cell peptides among the autoantigenic epitopes similar to *M. tuberculosis *proteins (Additional file 2). Our analysis for promiscuous epitopes revealed that although there were fewer promiscuous CD8 T cell epitopes (44), compared to CD4 T cell epitopes (242), most of them were from the metabolic proteins (26) (Additional file 1, 2).

Having recognized the possible autoreactive CD4 and CD8 T cell epitopes, we next addressed whether the host antigens containing cross-reactive epitopes had any known associations in autoimmune reactions. We identified that some of the host proteins (sharing similarity with *M. tuberculosis *proteins) were known to be implicated in diseases like MS, RA, Stiff-man syndrome, etc [4,19,40]. MS is an autoimmune demyelinating disorder that severely compromises patients. The precise etiology for the onset of the disease remains obscure. One of the hypotheses that explain the initiation of the disease is the occurrence of antecedent infections that ultimately lead to the pathogenesis of MS [4,41]. Intriguingly, individuals positive for HLA-DRB1*15:01 are known to be susceptible to both MS and tuberculosis [39,41]. One of the earlier observations suggested the presence of T cells in MS patients that cross-react to mycobacterial heat shock proteins (HSP) [18,19,21,41]. Our analysis showed several peptides from mycobacterial HSP60 (also known as HSP65) homologous to human HSP60 and it relatives, to bind to many different alleles (Table 1). One particular peptide, KPLVIIAEDVDGEALSTLVLN, promiscuously bound to many alleles including HLA-DRB1*15:01 with high affinity. This is suggestive of the fact that such cross-reactive epitopes may initiate the pathogenesis of MS. Another autoimmune disease that has been frequently associated with the occurrence of tuberculosis is RA [14,15,17]. Intriguingly, HSP60 of *M. tuberculosis *is also implicated in the pathogenesis of RA [14,15,17]. Our analysis also concurs with this hypothesis (Table 1). There are reports that suggest the presence of HSP60 reactive T cell clones which can also be reactive to the cartilage in the synovia of RA patients [14]. Hence, HSP60 cross reactive epitopes may play a significant role in the etiology of RA and MS. Further, we could also identify many T cell epitopes from different antigens like gephyrin, triosephosphate isomerase, etc., which are associated with Stiff-man syndrome, arthritis, etc. The putative autoimmune epitopes with the restricting alleles for known autoantigens that shared sequence similarity with *M. tuberculosis *proteins are listed in Table 1. The results discussed in the current section (Table 1) were solely derived from proteins known to be linked with autoimmune diseases [4,14-18,20]. However, other host antigens, whose putative T cell autoreactive epitopes are identified here, may be involved in the etiology of many other autoimmune diseases that need to be clinically verified.

**Table 1:** Putative CD4 T cell epitopes of established antigens involved in autoimmune diseases that share similarity with *M.tuberculosis *proteins.

Protein from ***M. tuberculosis***.	Disease	Homologous Human Proteins (known to be associated with autoimmunity)	Putative T cell epitope	HLA Class II Alleles
HSP60	Multiple Sclerosis [4,18,19,21]		IGAKLVQDVA	HLA DRB1* 01:01
		HSP60	EGMKFDRGYIS	HLA DRB1* 01:01, 03:01,04:01,07:01,11:01,15:01
			VAVKAPGFGD	HLA DRB1*15:01
			KPLVIIAEDVDGEALSTLVLN	HLADRB1*01:01,03:01,04:01,07:01,08:02,11:01,13:01,15:01
		cDNA FLJ54912	EGMKFNRGYIS	HLA DRB1*01: 01, 04: 01, 07: 01, 01: 01, 13: 01, 15: 01
			KPLVIIAEDVDGEALSTLV	HLA DRB1*01: 01, 03: 01, 04: 01, 07: 01, 08: 02, 11: 01,15: 01
		60 kDa chaperonin (Fragment) [HSPD1]	EGIKFDRGYIS	HLA DRB1*01: 01, 03: 01, 04: 01, 07: 01, 08: 02, 11: 01, 13: 01, 15: 01
			LKFDRGYVS	HLA DRB1*01: 01, 03: 01, 04: 01, 07: 01, 11: 01, 13: 01
	Rheumatoid Arthritis [14,15,17]	Putative uncharacterized protein HSPD1	GEALSTLVLN	HLA DRB1*01: 01
			KPLVIIAEDVDGEALSTLVLN	HLA DRB1*01: 01, 03: 01, 04: 01, 07: 01, 08: 02, 11: 01, 13: 01, 15: 01
		T-complex protein 1 subunit beta	LALVTGGEI	HLA DRB1*01: 01, 07: 01, 13: 01, 15: 01
		T-complex protein 1 subunit epsilon	LDKISDSVL	HLA DRB1*01: 01, 07: 01

Molybdopterin biosynthesis Mog protein	Stiff Man's Syndrome [40]	Gephyrin	LNLILTTGGTG	HLA DRB1*01: 01, 04: 01, 07: 01, 11: 01, 13: 01, 15: 01
		Highly similar to Gephyrin	GKTLIINLPGS	HLA DRB1*01: 01, 04: 01, 07: 01, 11: 01, 15: 01

Serine/threonine-protein kinase pknD	Paraneoplastic Limbic Encephalitis [49]	BR serine/threonine-protein kinase-2	HRDLKPENLLL	HLA DRB1*01: 01, 07: 01, 13: 01

Isoleucyl-tRNA synthetase	Arthritis [50,51] SLE [51]	Isoleucyl-tRNA synthetase	GLPHYGHIL	HLA DRB1*01: 01, 15: 01
	Interstitial lung disease [50,52]			

## Disscusion

Molecular mimicry between antigenic determinants present in pathogenic organisms and host proteins could potentially trigger autoimmune reactions [42]. Interestingly, many mycobacterial antigens have been associated with autoimmune diseases [14,16,17]. This prompted us to investigate the occurrence of peptides in mycobacteria that share sequence similarity with human antigens; and identify T cell epitopes that probably would be responsible for autoimmunity.

Although, *in silico *tools have been used in the past to examine molecular mimics in other diseases [5]; the knowledge of such epitopes from mycobacteria still needs to be explored. Here, utilizing *in silico *methods, we have identified potential autoreactive CD4 and CD8 T cell epitopes that may act as molecular mimics and result in autoimmune response during *M. tuberculosis *infection. The following major findings have emerged from the present study: (i) there is an extensive number of potentially autoreactive CD4 and CD8 T cell epitopes that are similar to peptides of mycobacterial antigens; (ii) the majority of such epitopes are similar to the antigens from the metabolic proteins of mycobacteria; (iii) a considerable number of promiscuous CD4 T cell epitopes could be detected; (iv) some of the identified antigens were corroborated with established autoimmune diseases linked with mycobacterial infection, thus validating the approach. We believe that this study would be a suggestive starting point for future investigation that whether mycobacterial infections and molecular mimics may elicit T cell autoimmune reactions.

Autoimmune reactions occur as a consequence of the breakdown of self-tolerance. Even though the immune system has central and peripheral tolerance mechanisms to deter the presence of autoreactive T cells, the very occurrence of autoimmune diseases signifies that this may not totally eliminate the presence or detrimental activity of host reactive T cells [6]. Autoimmune diseases develop as a result of multifactorial influences like genetic, hormonal, and environmental factors [43,44]. One of these key elements that substantially influence the development of autoimmunity is the occurrence of antecedent infections [45]. Almost every autoimmune disease investigated is assumed to be linked to one or more such infections. One of the classical evidences arguing for this hypothesis is the autoimmune acute rheumatic fever, which is associated with the infection with *Streptococcus pyogenes*. Molecular resemblance between the bacterial M-protein and human glycoproteins results in a breakdown of self-tolerance [46]. The molecular mimicry hypothesis proposes that shared epitopes between the host and pathogen can break tolerance and elicit autoreactivity. The degeneracy of antigen recognition by the T cell receptor may also help in such cross-reactivity [47]. Similarly, pathogen specific antibodies also can cross-react with host proteins [47]. Hence, molecular mimicry and consequent epitope spreading is now a generally accepted phenomenon influencing autoimmune reactions [3,42,45,47]. The idea of molecular mimicry was strongly put forward by Fujinami and Oldstone, where they argued that molecular mimicry could contribute pathogenesis of MS [48]. The criteria for this mechanism includes that the pathogen must be associated with the onset of the autoimmune reactions, the antigens from the pathogen must provoke an immune response that cross-reacts with host proteins. Further, the cross-reactive epitopes should induce disease, if tested in an animal model [45]. There are many reports that act as evidences to satisfy each of these criteria [2,7,44,45].

The presentation of certain antigenic epitopes (that are mimics of host antigens) to T cells by the pathogen encountered "TLR licensed" APCs may initiate the autoreactive responses [6]. Thus, homologous antigens from pathogens can potentially "revive" the otherwise non-responding autoreactive T cells. The inflammatory cytokines present during such priming may also imprint tissue migratory properties. When such autoreactive T cells come across the "cognate" host antigens, they will destruct tissues by their effector mechanisms. Hence, infection not only activates the autoreactive T cells but also may empower them to migrate to distant tissues thus initiating a process that ultimately escalates in to a full-bloomed disease. Tuberculosis has been associated with autoimmune reactions [13,16]. Classical studies have demonstrated the occurrence of mycobacterium reactive T cells that cross-react to antigens associated with MS, RA, etc [18-21]. Interestingly, in many cases the antigen was found to be HSP60 [14,18,20]. The present study also corroborates with these findings. In addition, our extensive comparative analysis of the proteomes of *M. tuberculosis *and humans followed by T cell epitope identification has revealed many more such possible target proteins and their putative epitopes. In the present scenario, where identifying the etiology of autoimmune diseases remains a great challenge, we believe that the outcome of the present study would open up extensive future investigations into molecular basis of possible T cell autoimmune reactions during mycobacterial infections.

## Conclusions

In essence, this study indicates the existence of considerable number of potential cross-reactive T cell epitopes between *M. tuberculosis *and the human proteome, which may elicit molecular mimicry and result in autoimmune responses during *M. tuberculosis *infection. Some of the epitopes were promiscuously binding to predominantly occurring HLA alleles and corroborated well with established autoimmune diseases. The identified target proteins and their putative T cell epitopes may have significant implications that will open up extensive investigations in understanding the molecular basis of autoimmune reactions during *M. tuberculosis *infection.

## Competing interests

The authors declare that they have no competing interests.

## Authors' contributions

JA and UG designed the study; SBC, PKR and SP performed the experiments; UG and SBC analyzed the data; JA, UG and SBC wrote the manuscript. All the authors read and approved the final manuscript.

## Supplementary Material

Additional file 1 **List of putative cross reactive epitopes from human proteome restricted to HLA class II alleles**. Data sets represent a list of human peptide regions showing sequence similarity with *M. tuberculosis*. These peptides were assessed for binding to predominant HLA class I and HLA class II alleles. Based on IC_50 _values, they were classified as strong binders (IC_50 _≤ 500), weak binders (500 ≤ IC_50 _≤ 5000) and non-binders (IC_50 _≥ 5000). Peptides binding to more than three different alleles were considered as promiscuous. Click here for file

Additional file 2 **List of putative cross reactive epitopes from human proteome restricted to HLA class I alleles**. Data sets represent a list of human peptide regions showing sequence similarity with *M. tuberculosis*. These peptides were assessed for binding to predominant HLA class I and HLA class II alleles. Based on IC_50 _values, they were classified as strong binders (IC_50 _≤ 500), weak binders (500 ≤ IC_50 _≤ 5000) and non-binders (IC_50 _≥ 5000). Peptides binding to more than three different alleles were considered as promiscuous. Click here for file
